# Acidity Constants of Boronic Acids as Simply as Possible: Experimental, Correlations, and Prediction

**DOI:** 10.3390/molecules29112713

**Published:** 2024-06-06

**Authors:** Andrzej Sporzyński, Agnieszka Adamczyk-Woźniak, Dorota Zarzeczańska, Jan T. Gozdalik, Paulina Ramotowska, Wiktoria Abramczyk

**Affiliations:** 1 Faculty of Agriculture and Forestry, University of Warmia and Mazury, Oczapowskiego 2, 10-719 Olsztyn, Poland; abramczyk.wiktoria6@gmail.com; 2 Faculty of Chemistry, Warsaw University of Technology, Noakowskiego 3, 00-664 Warsaw, Poland; agnieszka.wozniak@pw.edu.pl (A.A.-W.); jgozdalik@yahoo.com (J.T.G.); 3 Faculty of Chemistry, University of Gdańsk, Wita Stwosza 63, 80-308 Gdańsk, Poland; dorota.zarzeczanska@ug.edu.pl (D.Z.); paulina.ramotowska@wp.pl (P.R.)

**Keywords:** boronic acid, Lewis acid, acidity constant, Hammett equation

## Abstract

The wide use of boronic compounds, especially boronic acids and benzoxaboroles, in virtually all fields of chemistry is related to their specific properties. The most important of them are the ability to form cyclic esters with diols and the complexation of anions. In both cases, the equilibrium of the reaction depends mainly on the acidity of the compounds, although other factors must also be taken into account. Quantification of the acidity (p*K*_a_ value) is a fundamental factor considered when designing new compounds of practical importance. The aim of the current work was to collect available values of the acidity constants of monosubstituted phenylboronic acids, critically evaluate these data, and supplement the database with data for missing compounds. Measurements were made using various methods, as a result of which a fast and reliable method for determining the p*K*_a_ of boronic compounds was selected. For an extensive database of monosubstituted phenylboronic acids, their correlation with their Brønsted analogues—namely carboxylic acids—was examined. Compounds with ortho substituents do not show any correlation, which is due to the different natures of both types of acids. Nonetheless, both meta- and para-substituted compounds show excellent correlation. From a practical point of view, acidity constants are best determined from the Hammett equation. Computational approaches for determining acidity constants were also analyzed. In general, the reported calculated values are not compatible with experimental ones, providing comparable results only for selected groups of compounds.

## 1. Introduction

Arylboronic acids and their derivatives are an important group of compounds due to their broad applications in organic synthesis, catalysis, supramolecular chemistry, and materials engineering [1,2,3,4]. These compounds have been known for over 100 years, but interest in them increased after the discovery of new potential areas of their use. The most important applications of boronic acids are the synthesis of biaryl compounds in the Suzuki–Miyaura reaction (Nobel prize in 2010) [5], as the molecular receptors of sugars [6], as Covalent Organic Frameworks (COFs) [7], and as biologically active compounds [8,9].

Designing new compounds with predictable properties involves knowledge of their basic physicochemical quantities related to specific applications. One of the most important parameters of boronic acids is their acidity. This is the basic parameter determining the equilibrium of formation of cyclic diols with polyols. This reaction is, in many cases, the basis of biological action due to inhibiting pathogen enzymes. Another important area connected with acidity is the field of molecular receptors [10]. Finally, the catalytic activity of boronic acids is also closely related to their acidity [11,12].

In most cases, boronic acids have the character of Lewis acids. Due to the electron deficiency on the boron atom, they form complexes with Lewis bases (e.g., the hydroxyl anion). Equilibrium in water is described by Equation (1):

RB(OH)_2_ + 2 H_2_O = RB(OH)_3_^−^ + H_3_O^+^ (1)

The thermodynamic acidity constant based on this reaction is defined by Equation (2):

(2) $$K_{a}=\frac{a_{{RB\left(OH\right)}_{3}^{-}}\cdot a_{H_{3}O^{+}}}{a_{{RB\left(OH\right)}_{2}}},\;\;\;\;\;\;\;\;\;pK_{a}=-logK_{a}$$

In practice, approximate equilibrium constants based on molar concentrations are used (3):

(3) $$K_{a}^{\prime }=\frac{\left[{RB\left(OH\right)}_{3}^{-}\right]\cdot [H_{3}O^{+}]}{\left[{RB\left(OH\right)}_{2}\right]}$$

The dependence of the activity on the concentration is given by Equation (4):

(4) $$a_{i}=c_{i}\cdot \gamma_{i}$$

where $\gamma_{i}$ is the activity coefficient. The values of activity coefficients depend on the ionic strength of the solution, i.e., on the presence of all types of ions. For dilute solutions, the values of the activity coefficients are close to 1. An alternative is to use an electrolyte with a much higher concentration than the reagents involved in the reaction, for example, 0.1 M KCl. In this situation, despite titrating the analyte solution, no significant changes in the ionic strength of the solution are observed. In further considerations, we will deal with the equilibrium concentration constant (3), omitting the index for simplicity.

Despite the numerous Lewis acidity measurement methods, there is no universal Lewis acidity scale. IUPAC defines Lewis acidity as the thermodynamic tendency for Lewis pair formation. This strength was recently specified as “global Lewis acidity” [13]. It can be measured by simple anion (e.g., OH^−^ or F^−^) affinity. According to this approach, the Lewis acidity of a boronic acid in aqueous solution is given by the Equation (2). Reaction with a fluoride anion, applied in F^−^ sensors [14], is more complex due to the further exchange of OH^−^ for F^−^, leading to the formation of the anion PhBF_3_^−^ [15,16].

Experimentally, Lewis acidity is usually evaluated by the effect on the Lewis base molecule, and is called “effective Lewis acidity” [13]. It corresponds to the changes in the physicochemical properties of a probe Lewis base upon binding of the Lewis acid. It needs to be emphasized that, for a given Lewis acid, the electronic, steric, and solvation effects depend on the type of probe Lewis base molecule.

One of the most often applied Lewis acidity parameters is the acceptor number (AN), proposed in 1975 by Gutmann [17,18] and subsequently applied to organoboron compounds by Beckett et al. [19]. It involves determining the shift of the ^31^P NMR signal after the formation of a complex between triethylphosphine oxide (TEPO) and a Lewis acid. Taking into account the equilibrium nature of this reaction, the method was further modified by extrapolating the data to an infinite excess of the organoboron compound [20].

The Gutmann–Beckett method works well for determining the relative acidities of compounds containing one acidic center, e.g., boron atoms in boronate esters or triorganoboranes. In the case of boronic acids, the presence of hydroxyl groups allows processes other than the simple formation of a Lewis pair. Examples include systems containing amines. In the case of sufficiently basic amines, the reaction, regardless of the molar ratio of the reactants, leads to the formation of a stable complex with boroxine, (ArBO)_3_*RNH_2_. This reaction was first described by Yabroff and Branch in 1933 [21]. In 2005, the equilibrium in solutions was examined and the structure of the crystalline complex was confirmed [22]. This reaction was described again in 2006, and the authors called it “ligand-facilitated trimerization” [23].

The application of the Gutmann–Beckett method to studying the catalytic activity of boronic acids was investigated by Diemoz and Franz [24]. They found that the formation of a hydrogen bond is the predominant mode of interaction for boronic acid with TEPO, and the determined AN values correlate better with the catalytic activity in the Friedel–Crafts reaction than the p*K*_a_ values of the acids. Recently, Shmakov et al. published a review in which methods for determining the Lewis acidity of organoboron compounds in terms of their catalytic activity have been summarized and compared [11]. Correlation between catalytic activity and Lewis acidity was also discussed by Moran et al. [12].

Considering the described possibilities of determining the acidities of organoboron compounds, it seems that the dissociation exponent (p*K*_a_) remains both the simplest as well as the most universal tool for determining the acidic characters of boronic acids.

This article has the following objectives:

- Collecting acidity constant data for monosubstituted phenylboronic acids with simple substituents;
- Checking correlations with corresponding compounds (carboxylic acids);
- Checking correlations with data for solvents other than water;
- Selection of the simplest methods for determining p*K*_a_;
- Performing measurements for a series of missing compounds and assessing their accuracy;
- Correlation checking using the above results as a uniform database.

## 2. Results and Discussion

### 2.1. Correlation of Acidity: Boronic vs. Benzoic Acids

Carboxylic acids have the character of Brønsted acids (Equation (5)).

RCOOH + H_2_O = RCOO^−^ + H_3_O^+^ (5)

Despite their different nature of acidity, their equilibrium constant is defined by a similar formula as for boronic acids (6):

(6) $$K_{a}=\frac{\left[{RCOO}^{-}\right]\cdot [H_{3}O^{+}]}{\left[RCOOH\right]}$$

For both types of acids, the values of their acidity constants are influenced by the substituents in the phenyl ring. In the case of the presence of substituents at the ortho position, both steric factors as well as the possibility of specific chemical interactions of substituents with the neighboring COOH or B(OH)_2_ group obviously display a significant impact. Correlations between carboxylic and boronic acidity appeared previously in the literature, but the data concerned a small number of acids. Therefore, a broader examination of the correlation between both types of compounds is fully justified. It is worth noting that, while there are relatively few data on the acidity constants of boronic acids, for carboxylic acids, there are extensive databases with analysis of the values (e.g., [25,26]). What is more, for many boronic acid acidity studies, there is no detailed description of the applied experimental method or conditions.

For further analyses, the values of acidity constants for monosubstituted phenylboronic acids and their analogues—benzoic acids—were collected (Table 1).

The lack of values for several substituents shows that, even for such typical substituents as halogens or hydroxyl groups, many acidity constant values of the phenylboronic acids are missing.

**Figure 1:**
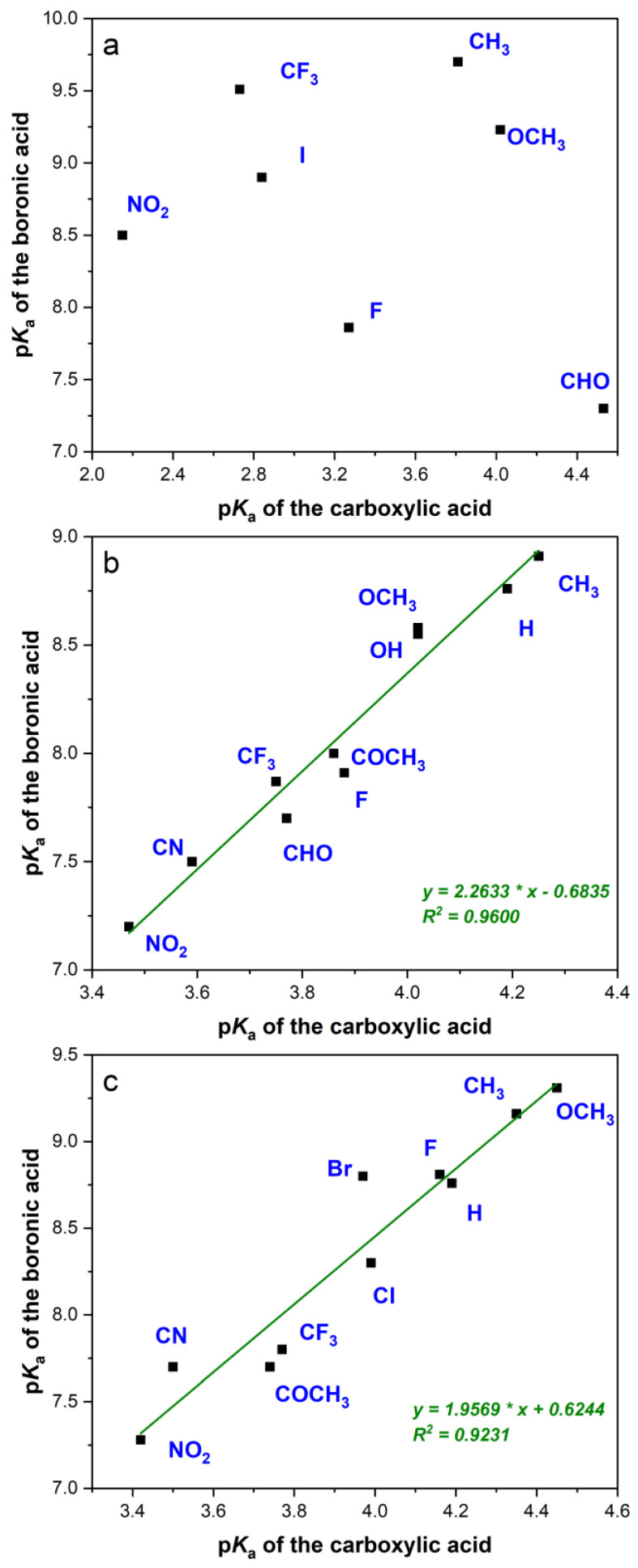
Correlation of the p*K*_a_ values of monosubstituted phenylboronic acids with benzoic acids: (**a**) ortho-, (**b**) meta-, (**c**) para-substituted compounds.

Correlations between available data for boronic and carboxylic acids were made separately for the ortho, meta, and para positions and are shown in Figure 1.

As expected, compounds with substituents at the ortho position show no correlation. This is due to possible intramolecular interactions of both the acidic and the basic forms of both species with the neighboring functional group. These interactions are dissimilar mostly due to different structures of the ionized forms—for carboxylic acids it is a planar structure, for boronic acids—a tetrahedral one.

In the case of boronic acids with substituents at the ortho position, the electronic effects are similar to those at the para position, but the acidity is additionally influenced by the interactions between the B(OH)_2_ group and the neighboring substituent. Two cases can be distinguished here:

1. The substituent does not form intramolecular hydrogen bonds with the B-OH group. In this case, the steric hindrance makes it difficult to form a tetrahedral form, and the acidity of the compound is lower than that of the corresponding para isomer. This can be observed for such substituents as CH_3_, NO_2_, and OCF_3_, and the reduction in acidity is approximately by one p*K*_a_ unit. A much stronger reduction occurs in the case of the bulk CF_3_ group—here, the change in p*K*_a_ is over 2 p*K*_a_ units. It is worth noting that the steric effect is opposite to that observed for carboxylic acids, where the acidity of the ortho isomer is higher than that of the para isomer.
2. The substituent forms intramolecular bonds with the B-OH group. This occurs in the case of such substituents as F, CHO, and OR [54], and the acidity of the ortho isomer is higher than that of the para isomer by from 0.3 to 0.9 units. The increase in acidity can be explained by the stabilization of the tetrahedral form B(OH)_3_^−^, which has a more favorable hydrogen bond geometry than in the case of the planar B(OH)_2_ group.

### 2.2. Hammett Equation

A more convenient method than comparing individual boronic and carboxylic acids is the Hammett Equation (7):

(7) $$logK_{X}-logK_{0}=\rho \cdot \sigma$$

where *K*_0_ is the acidity constant for unsubstituted benzoic acid, *K*_X_ is the constant for an acid with X substituent in the benzene ring, ρ is a constant for a given reaction under the given set of conditions, and σ is a constant characteristic for group X at a given (meta or para) position [55]. The Hammett equation describes electronic effects, and in its basic form it does not describe the effects of substituents at the ortho position. A positive value of the σ factor indicates an electron withdrawing group and a negative value an electron donating group. Although this equation was introduced for benzoic acids, being Brønsted acids, the acidity of both benzoic and arylboronic acids is determined by the electron density in their aromatic ring. Considering the limited amount of data in the literature on the acidity of boronic acids, compared to a very extensive database for benzoic acids, correlations between these two classes of compounds may be a convenient method for estimating the p*K*_a_ values of boronic acids.

**Figure 2:**
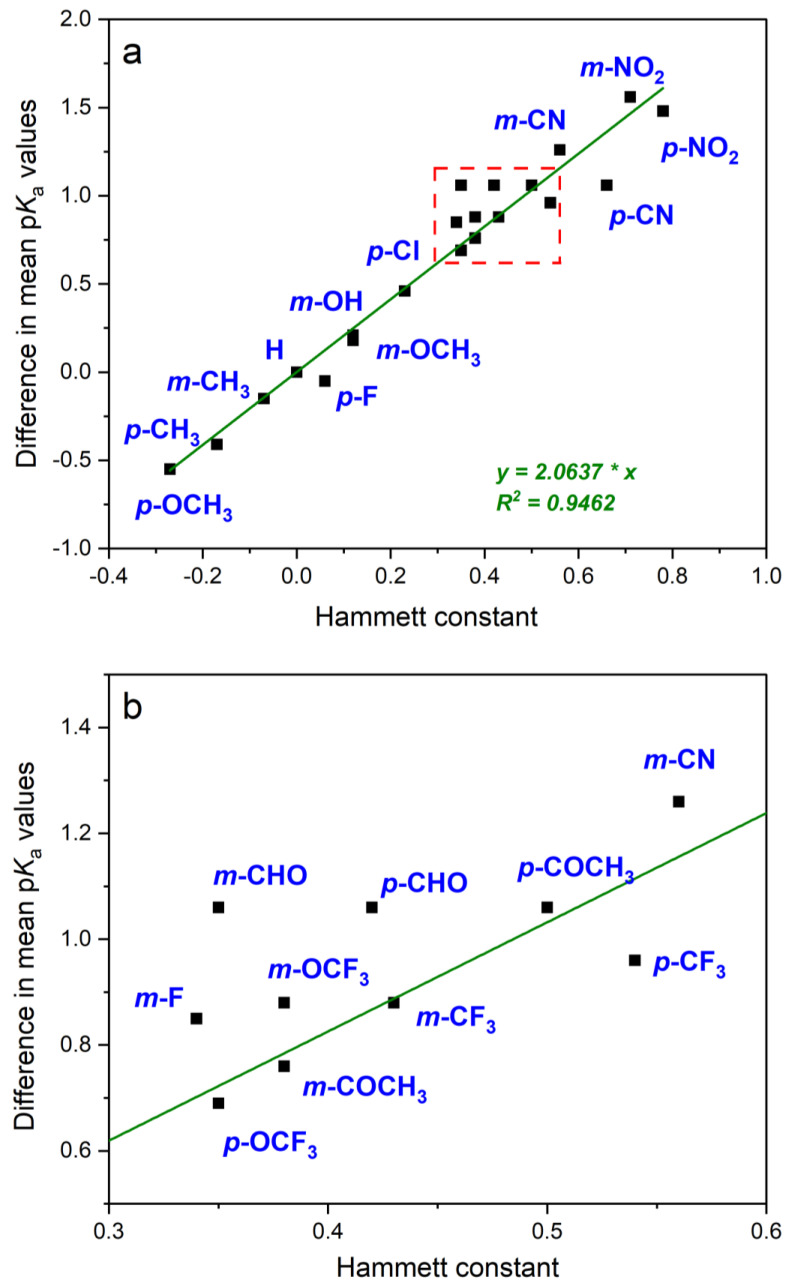
Plot of (p*K*_0_ − p*K*_x_) vs. substituent constant ($\sigma_{X}$ for meta- and para-monosubstituted phenylboronic acids). p*K*_0_ = 8.76 and p*K*_x_ data are the mean values from the data in Table 1. (**a**): All the data, (**b**): inset of the plot.

The reaction constant ρ for the dissociation of benzoic acid in an aqueous solution at 25 °C is 1.00. The Hammett equation describes well the effects of substituents for various aromatic systems. Once a set of σ values was obtained, ρ values could be calculated for other reactions. In practice, at least four well-spaced values are used to calculate ρ [56]. From the data collected in Table 1, the reaction constant for the dissociation of phenylboronic acids can be determined from the transformed Equation (7). The values of the substituent constants were taken from the paper [27], and the average p*K*_a_ values from Table 1 were applied to them. The result is shown in Figure 2.

The data presented in Figure 2 show good correlation. It is worth emphasizing that the data collected in Table 1 come from various sources and have been determined using different methods, and in many cases, there are no detailed descriptions of the experimental conditions. This results in significant differences in published values. The Hammett reaction constant determined from these data is equal to 2.06 and shows that the influence of substituents on the dissociation constants of phenylboronic acids is much greater than in the case of benzoic acids. The obtained value is close to the value of 2.146 given by Jaffé [55], calculated on the basis of works from the 1930s. The difference may be influenced by the fact that the constant values considered by Jaffé were determined not in pure water, but in a 25% aqueous ethanol solution.

Based on these data, the p*K*_a_ values for meta- and para-substituted phenylboronic acids can be calculated from Equation (8):

(8) $$pK_{X}=8.76-2.06\;\sigma_{X}$$

where 8.76 is the acidity exponent for phenylboronic acid (p*K*_0_), calculated as the mean value from data in Table 1. This equation makes it possible to determine the acidities of monosubstituted phenylboronic acids using extensive lists of Hammett constant values. For example, Hansch’s paper contains data for 530 substituents [27].

Although, for simple monosubstituted boronic acids, the above methods give good results, for many groups of compounds such an approach is not possible. Such systems include polysubstituted arylboronic acids, especially those with substituents at the ortho position, as well as larger organic molecules with incorporated boronic groups.

### 2.3. Other Solvents

The values of acidity constants are determined experimentally using various methods. The most used are potentiometric and spectrophotometric titration. Usually, values for dilute aqueous solutions determined by various methods do not differ significantly from each other. For instance, for 15 fluoro-substituted phenylboronic acids, differences between the data obtained by abovementioned methods do not exceed 0.08 units of p*K*_a_ (mean value 0.045) [38]. A similar compatibility was obtained by comparing data for isomeric trifluoromethyl- [52] and trifluoromethoxyphenylboronic acids [53]. Both methods give results of similar accuracy. In general, spectrophotometric determination is a more sensitive method, which is important in the case of substances poorly soluble in water, e.g., some drugs. Poor solubility in water is also a feature of most arylboronic acids [57]. Therefore, in many cases, organic solvents or their mixture with water are used, e.g., H_2_O/DMSO mixtures. However, the determination of acidity constants in such conditions requires a special approach. Tomsho et al. described the determination of p*K*_a_ in H_2_O/DMSO or in H_2_O/MeOH mixtures by UV/Vis titration, obtaining values of 9.1 and 9.2 depending on the DMSO concentration for PhB(OH)_2_ and 8.4 for 4-CF_3_C_6_H_4_B(OH)_2_ [58]. The values obtained in water are 8.76 (mean value) and 7.86 [52], respectively. Fini et al. showed that the p*K*_a_ values of substituted benzoic acid determined in the H_2_O/DMSO mixture correlate well with the values measured in water for groups of compounds with a similar substitution (ortho-substituted compounds treated separately from meta- and para-substituted ones) [59].

**Figure 3:**
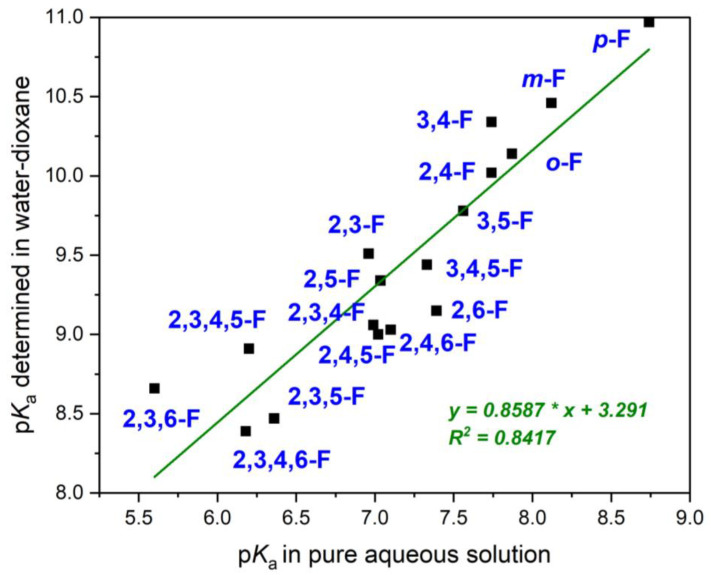
Correlation of p*K*_a_ values determined in water and water/dioxane (see Table 2).

In the case of boronic acids, it is difficult to find enough data to make such correlations. However, recently published works contain p*K*_a_ values of phenylboronic acids with fluorine substituents determined both in water [38] and a water/dioxane mixture [60]. The data are collected in Table 2 and the correlations of these values are shown in Figure 3.

The above comparison of values in different solvents shows a good correlation. It is worth emphasizing that data were compared for compounds differing in their number of substituents and their position in the ring. Correlations for monosubstituted compounds should give better results.

### 2.4. Determination of pK_a_ Values of New Compounds: Comparison of Experimental Methods

From a practical point of view, a method is needed that will allow the determination of acidity constants with sufficient accuracy using simple measurements and an uncomplicated calculation procedure. The duration of the measurement is also important: when titrating boronic acids with a strong base solution, the possibility of the hydrolysis of boronic acid protodeboronation should be taken into account [38].

In this part of this work, a group of monosubstituted boronic acids with Br, Cl, I, and CN substituents at the ortho, meta and para positions was selected. Acidity constants were measured for these compounds using potentiometric and spectrophotometric methods, exactly according to the procedure described for fluorine substituents [38]. As a result, a database of 24 new p*K*_a_ values was obtained by the same methods (spectroscopic and potentiometric 1) as described for fluoro-substituted compounds [38]. The p*K*_a_ values were obtained using a specialized software applying data analysis (see Section 3). The presented results were obtained by Kostrowicki and Liwo using specialized STOICHIO software [61,62,63], which analyzed measurement data based on the Marquardt algorithm. This method considers all measurement parameters and the resulting errors, such as the electromotive force, analyte and titrant volume, reagent concentrations and their purity, and electrode calibration parameters (*E*_o_ and *S*). The method of successive iterations is used to obtain the best fit of the curves obtained from the calculated values and measurement data.

As an alternative to the described procedure, it is possible to perform quick measurements by determining the p*K*_a_ as the pH value for half the volume of the titrant at the neutralization point. These data are included in Table 3 as “potentiometric 2”. The results are collected in the Table 3 together with the results for fluoro-substituted compounds from [38].

The introduction of a halogen atom into the ring of phenylboronic acids increases their acidity. A high increase is observed for meta substituents, due to the inductive effect and the lack of resonance effect. For para compounds, both effects act in the opposite direction, resulting in only a slight increase in acidity. The greatest changes are observed for ortho compounds. This is probably the effect of intramolecular hydrogen bond formation stabilizing the anionic form. In the case of iodine, the steric hindrance effect prevails, making it difficult to create the anionic form.

The comparison of results obtained by different methods showed that the spectrophotometric (S) and potentiometric (P1) ones give similar results with a root mean square (rms) of 0.14. The rms value of the compared potentiometric methods P1 and P2 is 0.18, which allows this method to quickly assess the acidity of boronic acids.

**Figure 4:**
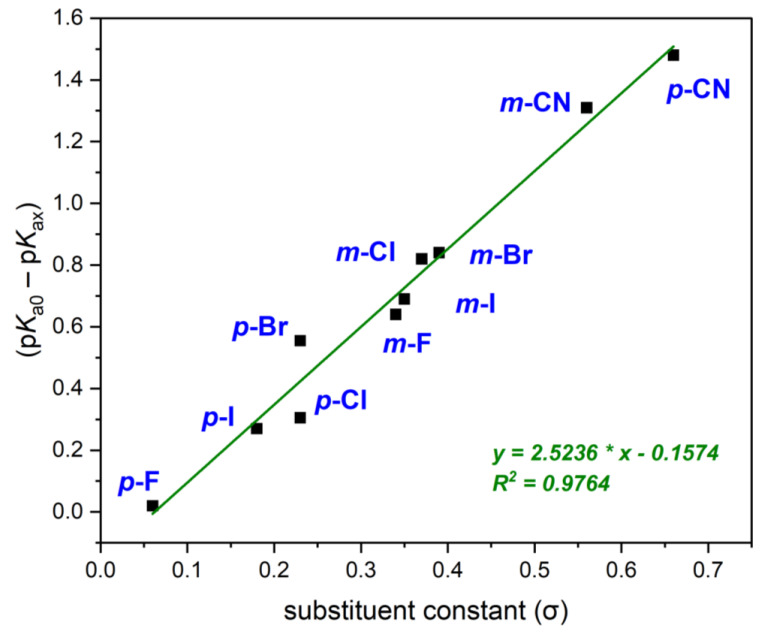
Plot of (p*K*_0_ − p*K*_x_) vs. substituent constant (σ) for meta- and para-monosubstituted phenylboronic acids obtained by spectroscopic and potentiometric (P1) methods (mean value).

The data collected in Table 3 are correlated with the Hammett σ constant in the same way as for the data from Table 1 and presented in Figure 4.

The presented data (Figure 4) show a very good correlation with the values of the Hammett constants. The p*K*_a_ values can be calculated from Equation (9):

(9) $$pK_{X}=8.92-2.52\;\sigma_{X}$$

### 2.5. Determination of pK_a_ by Calculations

**Figure 5:**
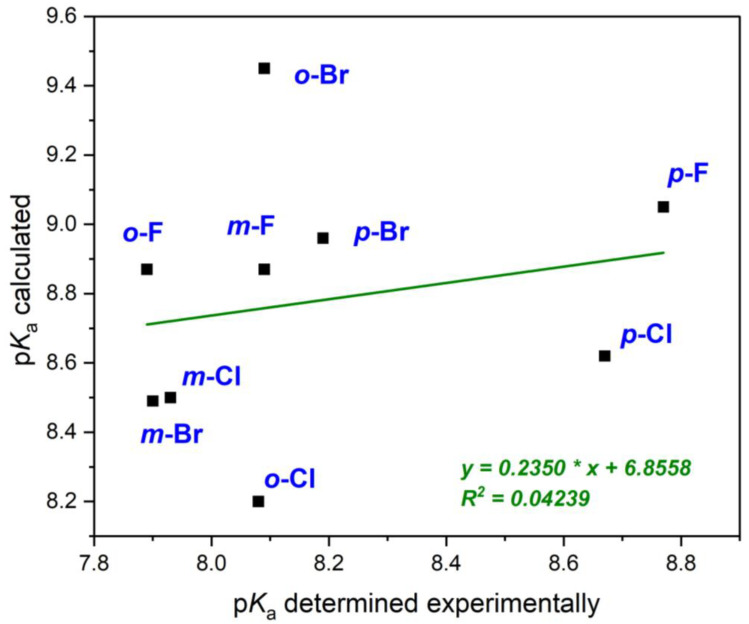
Calculated values [64] vs. experimental data (this work) for halogen-substituted phenylboronic acids.

In recent years, attempts were made to determine the p*K*_a_ of boronic acids via computational approach. The most extensive study includes calculations of acidity constants for 37 monosubstituted boronic acids [64]. The authors used the COSMO-RS model, in which the p*K*_a_ value was estimated from the free enthalpy of neutral and deprotonated boronic acid molecules. Unfortunately, the calculation results correlate poorly with experimental values. The differences in these values are ±1.5 p*K*_a_ units, which corresponds to the whole range for all monosubstituted phenylboronic acids. The authors explain the lack of correlation by discrepancies in the results obtained in different research groups. In the current work, we compared the calculation results of [64] with the experimental results collected for halogen substituents (Table 3, method S). The results are shown in Figure 5. 

The experimental and calculated data [64] show no correlation. The calculated values are much higher than the experimental ones. A similar result was obtained when only meta and para isomers were used for correlation (R^2^ = 0.23). Thus, the method cannot be used to predict the acidity of these compounds. Perhaps the reason for the observed discrepancy is the authors choice to consider a neutral molecule and the -B-O^−^ anion for Δ*G* calculations, while the equilibrium in aqueous solutions is described by Equation (1). Previously published computational results cover smaller groups of compounds and use other DFT methods with a different level theory, but the correlation is still weak [65,66].

## 3. Experimental

### 3.1. pKa Determination

The acid dissociation constants of the tested compounds were determined using two methods: potentiometric titration and spectrophotometric titration. The titrant in both experiments was a sodium hydroxide solution prepared by dissolving solid sodium hydroxide in water. The concentration of the prepared NaOH solution was determined by potentiometric titration with a standard 0.1 M hydrochloric acid solution. All experiments were carried out at 25 ± 1 °C.

### 3.2. Potentiometric Method P1

Potentiometric titrations were performed with a microtitration automatic Cerko-Lab system equipped with a Schott Blue line N16 pH electrode and a 1.0 mL Hamilton syringe. The pH glass-electrode was calibrated with four buffer solutions. The resolution of the voltage measurements was <0.1 mV. The compounds were dissolved in an aqueous solution with a constant ionic strength (0.1 M KCl). The concentrations of the boronic acid solutions were in the range of 2 × 10^−3^–8 × 10^−4^. Potential was recorded every 30 s. The dissociation constant values obtained with potentiometric method P1 were calculated using a STOICHIO version CV EQUID computer program which uses the non-linear least-squares Gauss–Newton–Marquardt method for fitting procedure [61,62,63].

### 3.3. Potentiometric Method P2

The pH glass electrode was calibrated with two buffer solutions (pH 4 and 7). A sample of boronic acid (0.03–0.05 g) was dissolved in 0.05 M aqueous KCl solution. After the complete dissolution of the sample, it was titrated with 0.05 M aq. NaOH. The inflection point on the curve corresponding to the neutralization point was determined from the derivative plot calculated as ΔpH/Δ*V*. The p*K*_a_ value was determined from the pH = f(*V*) curve as the pH value corresponding to the pH value for half the volume of the neutralization point.

### 3.4. Spectrophotometric Method

Spectrophotometric titrations were performed on a Perkin Elmer UV-Vis spectrophotometer Lambda 650, using quartz cuvettes of a 1 cm light pathlength. Spectrophotometric titrations were performed under conditions analogous to those used for the potentiometric measurements. For spectral changes presented as a *A* = f (pH) dependence, a correction to the dilution of the analyte was considered during the measurement. All measurements were performed at 298 K. The values obtained with the spectrophotometric method were calculated with the Henderson–Hasselbach equation implemented into Origin Lab v.2023 software. It is based on the change in the absorption intensity as a function of pH of the solution [67].

## 4. Conclusions

The p*K*_a_ values of boronic acids available in the literature are incomplete and vary widely due to various methods and conditions of their determination. Therefore, the use of correlation equations enabling the calculation of these values from data for other groups of compounds seems fully justified. Comparison of the p*K*_a_ values of monosubstituted phenylboronic and benzoic acids showed a good correlation for meta and para isomers. This proves that, although these two types of acids have different characters of acidity, in both cases, the p*K*_a_ value depends on the influence of the substituent in the benzene ring. Ortho isomers do not show any correlation due to the dominant steric effect and the possibility of intramolecular interactions that are different for both types of acids. The use of the Hammett equation allows for a good prediction of the p*K*_a_ value for meta and para phenylboronic acids.

Performing p*K*_a_ measurements for a series of new compounds completed the data for monosubstituted boronic acids. The comparison of values determined using different methods showed that the spectrophotometric and potentiometric methods give similar results. The fast potentiometric method gives similar results, but with a larger error.

Comparison of the so far reported results of theoretical calculations with experimental data in most cases does not show a satisfactory correlation.

## Figures and Tables

**Table 1:** Acidity (p*K*_a_ values) for selected monosubstituted benzoic and phenylboronic acids in water and substituent’s Hammett constants (σ).

X	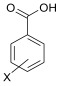 Benzoic			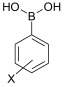 Boronic			Hammett Const. [27]	
H	4.21 [25], 4.16 [28]			8.9 [29], 8.7 [30], 8.64 [31], 8.86 [32],				
				8.72 [21], 8.90 [33], 8.70 [34]				
	*ortho*	*meta*	*para*	*ortho*	*meta*	*para*	*meta*	*para*
OH	2.85 [28]	4.09 [25]	4.59 [25]		8.55 [35]		0.12	−0.37
		3.94 [28]	4.48 [28]					
F	3.27 [36]	3.88 [25]	4.16 [25]	7.83 [37]	7.50 [37]	8.66 [37]	0.34	0.06
				7.89 [38]	8.09 [38]	8.77 [38]		
				7.85 [38]	8.15 [38]	8.71 [38]		
				8.7 [39]		8.6 [40]		
						9.1 [41]		
						9.0 [39]		
Cl	2.94 [42]	3.82 [42]	3.98 [42]			8.3 [29]	0.37	0.23
	3.01 [28]	3.70 [28]	4.00 [28]					
		3.84 [25]	4.00 [25]					
Br	2.85 [42]	3.81 [42]	4.00 [25]			8.8 [40]	0.39	0.23
	2.81 [28]	3.78 [28]	3.93 [28]					
			3.99 [25]					
I	2.84 [28]	3.79 [28]	3.98 [28]	8.9 [43]			0.35	0.18
		3.88 [25]	4.00 [25]					
CN	3.14 [44]	3.59 [25]	3.5 [25]		7.5 [39]	7.7 [39]	0.56	0.66
CH_3_	3.81 [28]	4.24 [28]	4.36 [28]	9.7 [29]	9.0 [29]	9.3 [29]	−0.07	−0.17
		4.27 [25]	4.35 [25]		9.00 [33]	9.26 [33]		
					8.74 [34]	8.95 [34]		
OCH_3_	4.02 [28]	3.92 [28]	4.41 [28]	9.0 [45]	8.7 [45]	9.3 [45]	0.12	−0.27
		4.12 [25]	4.49 [25]	9.0 [40]	8.5 [40]	9.3 [29]		
				9.0 [39]	8.7 [41]	9.32 [46]		
				9.7 [29]	8.4 [39]			
CHO	4.56 [47]	3.77 [48]		7.3 [45]	7.5 [45]	7.7 [45]	0.35	0.42
	4.5 [49]			7.31 [46]	7.8 [40]	7.6 [40]		
					7.80 [46]	7.80 [46]		
COCH_3_		3.86 [25]	3.74 [25]		8.0 [45]	7.7 [45]	0.38	0.50
					8.0 [40]	7.7 [40]		
NO_2_	2.17 [42]	3.45 [42]	3.44 [42]	ca. 8.5 [46]	7.1 [40]	7.4 [39]	0.71	0.78
	2.14 [28]	3.50 [25]	3.40 [25]		7.3 [46]	7.15 [46]		
CF_3_	2.73 [50]	3.75 [25]	3.77 [51]	9.58 [52]	7.88 [52]	7.39 [37]	0.43	0.54
		3.90 [50]	3.77 [50]	9.45 [52]	7.85 [52]	8.1 [39]		
				8.5 [39]	7.87 [37]	7.82 [52]		
					7.9 [39]	7.90 [52]		
OCF_3_				9.53 [53]	7.79 [53]	8.11 [53]	0.38	0.35
				9.49 [53]	7.96 [53]	8.03 [53]		

**Table 2:** p*K*_a_ values of fluoro-substituted phenylboronic acids in water (determined by spectrophotometric and potentiometric method at 25 °C) [38] and in water/dioxane mixture (1:1, 70 °C) [60].

Substituent	p*K*_a_ H_2_O (Spectr)	p*K*_a_ H_2_O (Pot)	p*K*_a_ H_2_O/Dioxane
2F	7.89	7.85	10.14
3F	8.09	8.15	10.46
4F	8.77	8.71	10.97
2,3F	6.99	6.93	9.51
2,4F	7.75	7.73	10.02
2,5F	7.06	7.01	9.34
2,6F	7.37	7.41	9.15
3,4F	7.74	-	10.34
3,5F	7.60	7.52	9.78
2,3,4F	6.97	7.01	9.06
2,3,5F	6.34	6.38	8.47
2,3,6F	5.60	-	8.66
2,4,5F	7.06	6.98	9.00
2,4,6F	7.10	-	9.03
3,4,5F	7.34	7.32	9.44
2,3,4,5F	6.23	6.17	8.91
2,3,4,6F	6.17	6.19	8.39

**Table 3:** Acidity constants for F-, Cl-, Br-, I-, and CN-substituted phenylboronic acid in water at 25 °C (data obtained in this work except for F substituent).

Substituent	Position	p*K*_a_			Hammett Constant σ
		Spectrophotometric S	Potentiometric P1	Potentiometric P2	
F *	*ortho*	7.89 ± 0.01	7.85 ± 0.07	-	-
	*meta*	8.09 ± 0.01	8.15 ± 0.11	-	0.34
	*para*	8.77 ± 0.01	8.71 ± 0.10	-	0.06
Cl	*ortho*	8.08 ± 0.01	8.07 ± 0.02	7.96 ± 0.02	-
	*meta*	7.93 ± 0.02	7.95 ± 0.03	7.81 ± 0.01	0.37
	*para*	8.67 ± 0.04	8.24 ± 0.04	8.17 ± 0.02	0.23
Br	*ortho*	8.09 ± 0.03	8.18 ± 0.03	8.04 ± 0.03	-
	*meta*	7.90 ± 0.04	7.94 ± 0.03	7.77 ± 0.01	0.39
	*para*	8.19 ± 0.01	8.22 ± 0.05	8.19 ± 0.03	0.23
I	*ortho*	8.63 ± 0.03	8.51 ± 0.04	7.92 ± 0.02	-
	*meta*	8.13 ± 0.05	8.01 ± 0.03	7.79 ± 003	0.35
	*para*	8.56 ± 0.01	8.42 ± 0.05	8.06 ± 0.01	0.18
CN	*ortho*	7.01 ± 0.10	6.89 ± 0.08	-	-
	*meta*	7.50 ± 0.02	7.40 ± 0.04		0.56
	*para*	7.32 ± 0.04	7.24 ± 0.04		0.66

* Data from reference [38].

## Data Availability

Data are contained within the article.

## References

[1] Hall D.G., Hall D.G. (2011). Boronic Acids: Preparation and Applications in Organic Synthesis, Medicine and Materials.

[2] Nishiyabu R., Kubo Y., James T.D., Fossey J.S. (2011). Boronic acid building blocks: Tools for sensing and separation. Chem. Commun..

[3] Adamczyk-Woźniak A., Borys K.M., Sporzyński A. (2015). Recent Developments in the Chemistry and Biological Applications of Benzoxaboroles. Chem. Rev..

[4] Adamczyk-Woźniak A., Sporzyński A., Wythers M.C. (2018). Diboronic Acids and Their Derivatives: New Perspectives in Sensing and Materials’ Chemistry. Advances in Materials Science Research.

[5] Suzuki A. (2011). Cross-Coupling Reactions of Organoboranes: An Easy Way to Construct C-C Bonds (Nobel Lecture). Angew. Chem. Int. Ed..

[6] Whyte G.F., Vilar R., Woscholski R. (2013). Molecular recognition with boronic acids-applications in chemical biology. J. Chem. Biol..

[7] Ding S.-Y., Wang W. (2013). Covalent organic frameworks (COFs): From design to applications. Chem. Soc. Rev..

[8] António J.P.M., Russo R., Carvalho C.P., Cal P.M.S.D., Gois P.M.P. (2019). Boronic acids as building blocks for the construction of therapeutically useful bioconjugates. Chem. Soc. Rev..

[9] Dhawan B., Akhter G., Hamid H., Kesharwani P., Alam M.S. (2022). Benzoxaboroles: New emerging and versatile scaffold with a plethora of pharmacological activities. J. Mol. Struct..

[10] James T.D., Phillips M.D., Shinkai S., James T.D., Phillips M.D., Shinkai S. (2006). Boronic Acids in Saccharide Recognition.

[11] Shmakov M.M., Prikhod’ko S.A., Panchenko V.N., Timofeeva M.N., Bardin V.V., Parmon V.N., Adonin N.Y. (2024). The organoboron compounds: Their Lewis acidity and catalytic activity. Catal. Rev..

[12] Zhang S., Lebœuf D., Moran J. (2020). Brønsted Acid and H-Bond Activation in Boronic Acid Catalysis. Chem.–Eur. J..

[13] Erdmann P., Greb L. (2022). What Distinguishes the Strength and the Effect of a Lewis Acid: Analysis of the Gutmann–Beckett Method. Angew. Chem. Int. Ed..

[14] Wu X., Chen X.-X., Song B.-N., Huang Y.-J., Ouyang W.-J., Li Z., James T.D., Jiang Y.-B. (2014). Direct sensing of fluoride in aqueous solutions using a boronic acid based sensor. Chem. Commun..

[15] Yuchi A., Sakurai J., Tatebe A., Hattori H., Wada H. (1999). Performance of arylboronic acids as ionophore for inorganic anions studied by fluorometry and potentiometry. Anal. Chim. Acta.

[16] Jańczyk M., Adamczyk-Woźniak A., Sporzyński A., Wróblewski W. (2012). Organoboron compounds as Lewis acid receptors of fluoride ions in polymeric membranes. Anal. Chim. Acta.

[17] Mayer U., Gutmann V., Gerger W. (1975). The acceptor number—A quantitative empirical parameter for the electrophilic properties of solvents. Monatshefte Chem./Chem. Mon..

[18] Gutmann V. (1976). Empirical parameters for donor and acceptor properties of solvents. Electrochim. Acta.

[19] Beckett M.A., Strickland G.C., Holland J.R., Sukumar Varma K. (1996). A convenient n.m.r. method for the measurement of Lewis acidity at boron centres: Correlation of reaction rates of Lewis acid initiated epoxide polymerizations with Lewis acidity. Polymer.

[20] Adamczyk-Woźniak A., Jakubczyk M., Sporzyński A., Żukowska G. (2011). Quantitative determination of the Lewis acidity of phenylboronic catechol esters—Promising anion receptors for polymer electrolytes. Inorg. Chem. Commun..

[21] Yabroff D.L., Branch G.E.K. (1933). Addition Compounds of Phenylboric Acid with Bases. J. Am. Chem. Soc..

[22] Sporzyński A., Lewandowski M., Zarychta B., Zaleski J. (2005). Complexes of Benzeneboronic Acid and Triphenylboroxin with Amines. Pol. J. Chem..

[23] Iovine P.M., Fletcher M.N., Lin S. (2006). Condensation of Arylboroxine Structures on Lewis Basic Copolymers as a Noncovalent Strategy toward Polymer Functionalization. Macromolecules.

[24] Diemoz K.M., Franz A.K. (2019). NMR Quantification of Hydrogen-Bond-Activating Effects for Organocatalysts including Boronic Acids. J. Org. Chem..

[25] Ludwig M., Baron V., Kalfus K., Pytela O., Vecera M. (1986). Dissociation constants of substituted benzoic acids in water and in organic solvents. Collect. Czecholslovak Chem. Commun..

[26] Kortüm G., Vogel W., Andrussow K. (1961). Dissociation Constants of Organic Acids in Aqueous Solutions.

[27] Hansch C., Leo A., Taft W. (1991). A survey of Hammett substituent constants and resonance and field parameters. Chem. Rev..

[28] Vandenbelt J.M., Henrich C., Vanden Berg S.G. (1954). Comparison of pKá Values Determined by Electrometric Titration and Ultraviolet Absorption Methods. Anal. Chem..

[29] Wang X., Yue D., Lv E., Wu L., Qin W. (2014). Reporter-Free Potentiometric Sensing of Boronic Acids and Their Reactions by Using Quaternary Ammonium Salt-Functionalized Polymeric Liquid Membranes. Anal. Chem..

[30] Bosch L.I., Fyles T.M., James T.D. (2004). Binary and ternary phenylboronic acid complexes with saccharides and Lewis bases. Tetrahedron.

[31] Torssell K. (1957). Zur Kenntnis der Arylborsauren. 7. Komplexbildung Zwischen Phenylborsaure und Fruktose. Ark. Kemi.

[32] Branch G.E.K., Yabroff D.L., Bettman B. (1934). The Dissociation Constants of the Chlorophenyl and Phenetyl Boric Acids1. J. Am. Chem. Soc..

[33] Bektenova G.A. (2010). Ionization constants of boronic acids and their complexation with diols. Russ. J. Phys. Chem. A.

[34] Kajimoto O., Saeki T., Nagaoka Y., Fueno T. (1977). Temperature-jump rate studies of the association reactions of boric and benzeneboronic acids with hydroxide ion. J. Phys. Chem..

[35] Clear C.G., Branch G.E.K. (1938). The dissociation of hydrogen ions from the sulfates of aminophenylboronic acids. J. Org. Chem..

[36] Ibrahem I., Hammar P., Vesely J., Rios R., Eriksson L., Córdova A. (2008). Organocatalytic asymmetric hydrophosphination of alpha,beta-unsaturated aldehydes: Development, mechanism and DFT calculations. Adv. Synth. Catal..

[37] Yamamoto Y., Matsumura T., Takao N., Yamagishi H., Takahashi M., Iwatsuki S., Ishihara K. (2005). Fast trigonal/tetragonal interconversion followed by slow chelate-ring closure in the complexation of boronic acids. Inorganica Chim. Acta.

[38] Zarzeczańska D., Adamczyk-Woźniak A., Kulpa A., Ossowski T., Sporzyński A. (2017). Fluorinated Boronic Acids: Acidity and Hydrolytic Stability of Fluorinated Phenylboronic Acids. Eur. J. Inorg. Chem..

[39] Minkkilä A., Saario S.M., Käsnänen H., Leppänen J., Poso A., Nevalainen T. (2008). Discovery of Boronic Acids as Novel and Potent Inhibitors of Fatty Acid Amide Hydrolase. J. Med. Chem..

[40] Yan J., Springsteen G., Deeter S., Wang B. (2004). The relationship among pKa, pH, and binding constants in the interactions between boronic acids and diols—It is not as simple as it appears. Tetrahedron.

[41] Westmark P.R., Gardiner S.J., Smith B.D. (1996). Selective Monosaccharide Transport through Lipid Bilayers Using Boronic Acid Carriers. J. Am. Chem. Soc..

[42] Oshikawa T., Pochamroen S., Takai N., Ida N., Takemoto T., Yamashita M. (2002). Molecular Recognition and Resolution of Geometrical Isomers of Benzoic Acids by Brucine. Heterocycl. Commun..

[43] Al-Zoubi R.M., Marion O., Hall D.G. (2008). Direct and Waste-Free Amidations and Cycloadditions by Organocatalytic Activation of Carboxylic Acids at Room Temperature. Angew. Chem. Int. Ed..

[44] Widequist S. (1951). On the stability of the cyanobenzoic acids in aqueous solution. Ark. Kemi.

[45] Kilinc E. (2024). Resistance of Acetyl-, Formyl-, and Methoxy-Phenylboronic Acids to Boroxine Formation and Their Employment in Fluoride Determination of Dental Formulations and Beverages by Fluorescence Quenching. J. Appl. Spectrosc..

[46] Torssell K., McClendon J.H., Somers G.F. (1958). Chemistry of Arylboric Acids VIII. The Relationship between Physico-chemical Properties and Activity in Plants. Acta Chem. Scand..

[47] Tirouflet J. (1954). Cinetique de louverture et de la fermeture des cycles lactoniques—Influences structurales. 3. Lactonisation des acides ortho-methylolbenzoiques. Bull. Soc. Chim. Fr..

[48] Mock W.L., Morsch L.A. (2001). Low barrier hydrogen bonds within salicylate mono-anions. Tetrahedron.

[49] Pytela O., Kulhánek J. (2002). Ortho Effect in Dissociation of Benzoic Acids with Electron-Accceptor Substituents Using the AISE Theory; Relation to *para* Substitution and Solvent. Collect. Czechoslov. Chem. Commun..

[50] Boiadjiev S.E., Lightner D.A. (1999). Carboxylic acid ionization constants by 19F NMR spectroscopy. J. Phys. Org. Chem..

[51] Yao W., Yu Z., Wen S., Ni H., Ullah N., Lan Y., Lu Y. (2017). Chiral phosphine-mediated intramolecular [3 + 2] annulation: Enhanced enantioselectivity by achiral Brønsted acid. Chem. Sci..

[52] Gozdalik J.T., Marek P.H., Madura I.D., Gierczyk B., Popenda Ł., Schroeder G., Adamczyk-Woźniak A., Sporzyński A. (2019). Structures and properties of trifluoromethylphenylboronic acids. J. Mol. Struct..

[53] Adamczyk-Woźniak A., Gozdalik J.T., Kaczorowska E., Durka K., Wieczorek D., Zarzeczańska D., Sporzyński A. (2021). (Trifluoromethoxy)Phenylboronic Acids: Structures, Properties, and Antibacterial Activity. Molecules.

[54] Adamczyk-Woźniak A., Sporzyński A. (2020). The influence of ortho-substituents on the properties of phenylboronic acids. J. Organomet. Chem..

[55] Jaffé H.H. (1953). A Reëxamination of the Hammett Equation. Chem. Rev..

[56] Smith M.B., March J. (2007). March’s Advanced Organic Chemistry Reactions, Mechanisms, and Structure.

[57] Sporzyński A., Leszczyński P. (2017). Solubility of phenylboronic compounds in water. Mediterr. J. Chem..

[58] Tomsho J.W., Benkovic S.J. (2012). Examination of the reactivity of benzoxaboroles and related compounds with a cis-diol. J. Org. Chem..

[59] Fini A., De Maria P., Guarnieri A., Varoli L. (1987). Acidity Constants of Sparingly Water-Soluble Drugs from Potentiometric Determinations in Aqueous Dimethyl Sulfoxide. J. Pharm. Sci..

[60] Cox P.A., Reid M., Leach A.G., Campbell A.D., King E.J., Lloyd-Jones G.C. (2017). Base-Catalyzed Aryl-B(OH)_2_ Protodeboronation Revisited: From Concerted Proton Transfer to Liberation of a Transient Aryl Anion. J. Am. Chem. Soc..

[61] Kostrowicki J., Liwo A. (1990). Determination of equilibrium parameters by minimization of an extended sum of squares. Talanta.

[62] Kostrowicki J., Liwo A. (1984). DECFAM—A new computer oriented algorithm for the determination of equilibrium constants from potentionmetric and/or spectrophotometric measurements—I: Basic principles of the method and calculations of equilibrium concentrations. Comput. Chem..

[63] Kostrowicki J., Liwo A. (1984). DECFAM—A new computer oriented algorithm for the determination of equilibrium constants from potentiometric and/or spectrophotometric measurements—II: Methods based on analytical expressions. Comput. Chem..

[64] Kurtz D.A., Dhar D., Elgrishi N., Kandemir B., McWilliams S.F., Howland W.C., Chen C.-H., Dempsey J.L. (2021). Redox-Induced Structural Reorganization Dictates Kinetics of Cobalt(III) Hydride Formation via Proton-Coupled Electron Transfer. J. Am. Chem. Soc..

[65] Aggarwal V.K., Hall D.G., Hooper T.N., Ingleson M.J., Ishid N., Ishihara K., Jakle F., Johnson H.C., Lee S., Leonori D., Whiting E., Fernández A. (2013). Topic in Organometallic Chemistry 49—Synthesis and Applications of Organoboron Compounds.

[66] Kheirjou S., Abedin A., Fattahi A. (2012). Theoretical descriptors response to the calculations of the relative pKa values of some boronic acids in aqueous solution: A DFT study. Comput. Theor. Chem..

[67] Chylewska A., Jacewicz D., Zarzeczańska D., Chmurzyński L. (2008). Determination of dissociation constants for coordination compounds of Cr(III) and Co(III) using potentiometric and spectrophotometric methods. J. Chem. Thermodyn..

